# Heart rate variability in normal and pathological sleep

**DOI:** 10.3389/fphys.2013.00294

**Published:** 2013-10-16

**Authors:** Eleonora Tobaldini, Lino Nobili, Silvia Strada, Karina R. Casali, Alberto Braghiroli, Nicola Montano

**Affiliations:** ^1^Division of Medicine and Pathophysiology, Department of Biomedical and Clinical Sciences “L. Sacco,” L. Sacco Hospital, University of MilanMilan, Italy; ^2^Department of Neuroscience, Centre for Epilepsy Surgery “C. Munari,” Centre of Sleep Medicine, Niguarda Ca' Granda HospitalMilan, Italy; ^3^Department of Science and Technology, Science and Technology Institute, Federal University of São Paulo, São José dos CamposSao Paulo, Brazil; ^4^Institute of Cardiology of Rio Grande do Sul, University Foundation of CardiologyPorto Alegre, Brazil; ^5^Sleep Medicine Unit, Fondazione S. MaugeriVeruno, Italy; ^6^International Clinical Research Center, St. Anne University HospitalBrno, Czech Republic

**Keywords:** autonomic nervous system, heart rate variability, sleep, non-linear analysis, obstructive sleep apnea, insomnia, SUDEP

## Abstract

Sleep is a physiological process involving different biological systems, from molecular to organ level; its integrity is essential for maintaining health and homeostasis in human beings. Although in the past sleep has been considered a state of quiet, experimental and clinical evidences suggest a noteworthy activation of different biological systems during sleep. A key role is played by the autonomic nervous system (ANS), whose modulation regulates cardiovascular functions during sleep onset and different sleep stages. Therefore, an interest on the evaluation of autonomic cardiovascular control in health and disease is growing by means of linear and non-linear heart rate variability (HRV) analyses. The application of classical tools for ANS analysis, such as HRV during physiological sleep, showed that the rapid eye movement (REM) stage is characterized by a likely sympathetic predominance associated with a vagal withdrawal, while the opposite trend is observed during non-REM sleep. More recently, the use of non-linear tools, such as entropy-derived indices, have provided new insight on the cardiac autonomic regulation, revealing for instance changes in the cardiovascular complexity during REM sleep, supporting the hypothesis of a reduced capability of the cardiovascular system to deal with stress challenges. Interestingly, different HRV tools have been applied to characterize autonomic cardiac control in different pathological conditions, from neurological sleep disorders to sleep disordered breathing (SDB). In summary, linear and non-linear analysis of HRV are reliable approaches to assess changes of autonomic cardiac modulation during sleep both in health and diseases. The use of these tools could provide important information of clinical and prognostic relevance.

“But what interests me here is the specific mystery of sleep partaken of or itself alone, the inevitable plunge risked each night by the naked man, solitary and unarmed, into an ocean where everything changes, the colors, the densities, and even the rhythm of breathing, and where we meet the dead. What reassures us about sleep is that we do come out of it, and come out of it unchanged, since some mysterious ban keeps us from bringing back with us in their true form even the remnants of our dreams”

*Memories of Adrian, Marguerite Yourcenar, 1951*

## Introduction

The simple observation that human beings, mammals and other animal species spend about one third or more of their lifetime sleeping strongly suggest how fundamental the physiological process of sleeping is (Chou et al., 2003; Saper et al., 2005).

Despite the fact that a large amount of data has been published on the biological meaning and function of sleep, several key points still need to be clarified.

The sleep process is characterized by the activation of a number of cortical, subcortical and medullar neural circuits, which cooperate in order to control sleep according to hormonal changes (i.e., melatonin and orexin), local factors such as adenosine accumulation, circadian variations (i.e., dark-light cycles) and other unknown factors (Saper et al., 2005). A key role in the physiology of sleep is played by the autonomic nervous system (ANS), whose regulation modulates cardiovascular functions during sleep onset and the transition to different sleep stages.

The analysis of heart rate variability (HRV) has been widely used as a non-invasive and reliable tool to evaluate cardiovascular autonomic control in health and disease. The application of different tools, such as linear and non-linear analysis of HRV during different sleep stages provided fundamental insight on the physiological autonomic changes that characterize wake-to-sleep transition, sleep onset, and different sleep stages [rapid eye movement (REM) and non-REM sleep (NREM)]. In addition, different HRV tools revealed important modifications of autonomic cardiac control in different pathological conditions, such as insomnia, primary neurological sleep disorders, and sleep disordered breathing (SDB), a group of diseases associated with an alteration in the normal breathing during sleep (Parish and Somers, 2004; Nobili et al., 2011).

The present review will focus on the fundamental principles of sleep structure, the most used linear and non-linear analyses of HRV and the application of these tools to assess the autonomic cardiac control in normal and pathological sleep.

### Wakefulness-sleep transitions

Transition from wake to sleep is relatively rapid, considering that sleep onset is a process which lasts no more than one minute in human beings (Takahashi et al., 2010).

Interestingly, the switch from wake to sleep and among the different sleep stages is not monodirectional (i.e., from NREM, to REM), but, on the contrary, it oscillates from NREM to REM and vice versa, together with fast changes in electroencephalographic (EEG) cerebral waves and the occurrence of arousals (Saper et al., 2010).

The neural regulation of this process is very complex, counting several neural networks of neurons located both in the cortex and in the medulla. A key regulatory mechanism in the wake to sleep shift is the relation between hypothalamic and monoaminergic neurons. In fact, it has been shown that monoaminergic neurons in the pons project to noradrenergic neurons in the locus coeruleus and to dopaminergic and serotoninergic relay stations in the raphe (Saper et al., 2010). Wake period is characterized by firing activity of these monoaminergic neurons, which inhibit ventrolateral preoptic nucleus (VLPO) and neurons which regulate the transition to REM sleep; this mechanism is capable of limiting a direct transition from wake to REM sleep. On the contrary, sleep is characterized by an increased activity of VLPO neurons, which, in turn, inhibits monoaminergic neurons, and, consequentially, triggers the sleep onset. This regulatory mechanism is only one of the most important neural network involved in the wakefulness—sleep switch, and it is called the flip-flop switch model (Saper et al., 2010).

The full description of the neural networks regulating the shift from NREM to REM sleep and vice versa is out of the scope of this review.

### Sleep structure

Sleep macrostructure is characterized by two separate physiological stages, REM sleep and NREM sleep.

REM sleep, identified in 1953, is a physiological state that includes REM and low muscle tone. On the opposite, NREM sleep is usually divided into two states, light sleep (NREM 1 and NREM2, or N1 and N2) and deep sleep (NREM3, or N3, also called Slow Wave Sleep, SWS); during NREM sleep rapid eyes movements are absent while muscle tone is usually considerable. REM and NREM sleep are characterized by typical EEG features.

In fact, during wakefulness with closed eyes, on the EEG a typical rhythm, the alpha rhythm, becomes evident. Alpha rhythm is characterized by low voltage and high frequency waves, with a frequency band bounded between 8 and 12 Hz.

During doziness and sleep onset, cortical rhythm slows, alpha rhythm disappears and theta rhythm appears alongside the alpha; lower frequency (3.5–7.5 Hz) and higher voltage waves create theta rhythm; alpha disappears as sleep becomes deeper.

During N2, alpha rhythm completely disappears and theta rhythm is the dominant EEG oscillation. In addition, two peculiar EEG elements become evident at this time: spindles and K complexes. Spindles derive from bursts of brain activity and are characterized by high frequency waveform (12–14 Hz) lasting more than 0.5 s, while waves with a first negative-high voltage peak followed by slow positive complex and a second negative peak are called K complexes. Their rate of occurrence is roughly every 1–2 min.

The transition from N2 to N3 is associated with the appearance of delta waves, which have high voltage (greater than 75 μV) and low frequency (bounded between 0.5 and 3 Hz). N3 is the sleep stage of highest synchronization of neural activity in the brain, with low muscle activity and no REMs. From N3 to REM, delta waves disappear and high frequency and low voltage waves become predominant; complete muscle atonia and REMs are observed.

## Heart rate variability as a window over autonomic cardiovascular control

**Figure 1:**
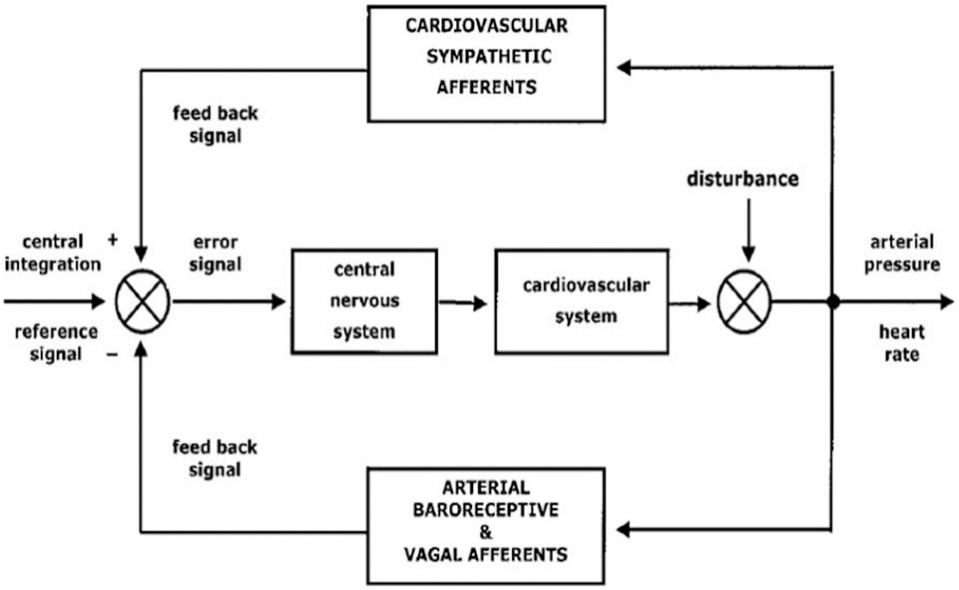
**A schematic representation of autonomic nervous system functions and its integration with internal and external stressor stimuli (from Montano et al., 2009; with permission)**.

The two branches of the ANS, sympathetic and parasympathetic nervous system, regulate visceral functions in order to maintain the homeostatic milieu of the body and to render the body able to react and to adapt to external and internal stressor stimuli (Malliani et al., 1991; Montano et al., 2009) (see Figure 1).

A very composite and interconnected regulating mechanisms operate at different levels, both central and peripheral, in order to coordinate ANS functions (Montano et al., 2009).

Within physiological conditions, the regulation of several fundamental visceral functions, such as cardiovascular, respiratory, and gastrointestinal systems, is based on the reciprocal activation of the two autonomic subsystems, the so-called “sympatho-vagal balance”: the activation of one branch, i.e., sympathetic outflow, is associated with a withdrawal of the other, i.e., parasympathetic drive, and vice versa (Malliani et al., 1998). This mechanism has been considered a key stone paradigm of the ANS function for many years; however, it has been suggested that the coactivation of both sympathetic and parasympathetic systems is not only physiologically possible, but it is also a rule in peculiar situations such as chemoreceptor reflexes (i.e., during apneas), exercise, and cold face immersion (Koizumi et al., 1982; Malliani and Montano, 2002; Paton et al., 2005).

These brief observations suggest the high degree of complexity of the ANS regulation, mainly due to the strong interconnection with several biological systems (central and peripheral nervous systems, immunity, inflammation, metabolism, hormones etc.) and to its multifaceted mechanisms of action of sympathetic and parasympathetic limbs (i.e., as antagonists or agonists).

For many years, several techniques have been developed for the assessment of ANS: (a) dosage of plasmatic and urinary catecholamines (Goldstein et al., 1983), which is nowadays not considered as a highly reliable index of sympathetic activity (Esler, 1993; Montano et al., 2009), (b) muscle sympathetic nerve activity (MSNA), a direct but invasive recording of sympathetic activity using a microneurography technique (Wallin and Charkoudian, 2007), (c) analysis of HRV, a non-invasive tool able to provide reliable information on sympathetic and parasympathetic oscillations of the heart period and arterial pressure time series, (d) more recent non-linear approaches based on entropy-derived measures and symbolic analysis of heart period time series (Porta et al., 2007a,b; Tobaldini et al., 2009).

### Linear analysis of HRV

A pioneering study by Lee and Hon first described significant changes in beat-to-beat intervals during fetal distress before evident changes in heart rate (HR) (Lee and Hon, 1965). During the next years, several evidences supported the hypothesis that rhythmical oscillations of both HR and blood pressure (BP) are indirect measures of sympathetic and parasympathetic modulation (Pagani et al., 1986; Malliani and Montano, 2002).

Therefore, HRV has been considered a non-invasive and reliable tool able to provide information on the sympathetic and parasympathetic modulation both in physiological and pathological conditions (Malliani et al., 1998; Montano et al., 2009). It is worth noting that HRV has been widely accepted not only as a tool to assess physiological autonomic functions, but also as a method able to provide important clinical information. Pioneering studies showed that, in post-myocardial infarction patients, total HRV was an independent predictor of mortality (Kleiger, 1987; Malik, 1989; Fei et al., 1996).

As stated above, HRV is based on the assessment of rhythmical oscillations embedded in heart period and blood pressure time series, which represent the sympathetic and parasympathetic modulations of cardiovascular function. Several computational methods have been validated, classically divided into non parametric (based on a simple algorithm, usually a fast Fourier transform) and parametric tools, such as autoregressive algorithm, in which spectral components are identified independently of preselected frequencies (Task Force of the European Society of Cardiology and The North American Society of Pacing and Electrophysiology, 1996).

On the heart period and blood pressure time series, three main components can be recognized: a very low frequency component (VLF), frequency band below 0.04 Hz; a low frequency component (LF), bounded between 0.04–0.15 Hz, and a high frequency component (HF, bounded between 0.15 and 0.4 Hz), synchronous with respiration. The VLF component is considered to be a marker of humoral and hormonal fluctuations; the LF component is discussed to represent a marker of both sympathetic and parasympathetic modulation while the HF component is considered a marker of vagal modulation.

In addition to the frequency band, each oscillation can be described in terms of amplitude, which can be expressed both in absolute (ms^2^ or mmHg^2^ for heart period and blood pressure time series, respectively) and in normalized units (nu). LFnu and HFnu represent the relative value of LF or HF with respect to the total variability, minus the VLF component (Malliani et al., 1991, 1998; Montano et al., 2009; Malliani and Montano, 2002).

It is worth noting that while HF oscillation is commonly accepted as a marker of parasympathetic modulation, the physiological meaning of the LF band is still debated. In fact, some authors have suggested that LF can be the result of sympathetic and parasympathetic modulation (Berntson et al., 1997; Eckberg, 1997; Billman, 2011, 2013), while, on the contrary, other experimental and clinical data support the hypothesis that LF is a marker of sympathetic modulation (Malliani et al., 1991, 1998; Montano et al., 2009).

### Non-linear analysis of HRV

Classic power spectral analysis of HRV is based on the assumption that heart period time series contain only linear and stationary dynamics; however, in the last years, increasing interest has been paid to non-linear dynamics that characterize autonomic cardiovascular control (Goldberger et al., 1988; Kaplan et al., 1991; Voss et al., 1995). Interestingly, it has been demonstrated that some non-linear parameters are better predictors of morbidity and mortality than standard linear spectral parameters in cardiac patients (Mäkikallio et al., 2001). Although several non-linear methods have been developed, in the present review we will briefly present entropy-derived measures, which have been recently applied for the assessment of autonomic cardiovascular complexity in physiological and pathological sleep.

#### Entropy-derived measures

Physiologically, biological variables are controlled by the interaction of several systems, which actively interact with each other as agonists or antagonists at different time scales. In this perspective, beat-to-beat regulation is under the influence of sympatho-vagal balance, central oscillators, reflexes circuits such as baroreflex and chemoreflex control, sympatho-sympathetic reflexes, molecular, and hormonal regulation. All these mechanisms are responsible for HRV complexity (Goldberger et al., 1988; Kaplan et al., 1991).

During aging and pathological situations, one of these mechanisms may become predominant, with a decrease or an inhibition of the others, thus leading to a decrease of complexity of HRV and to a simplification of cardiovascular regulation. If this is the case, plasticity of cardiovascular control is strongly impaired and the ability of the system to respond to internal and external stressor stimuli is significantly damaged. Interestingly, some complexity indices are powerful predictors of mortality in high-risk patients (Voss et al., 1996; Huikuri et al., 2003; Clariá et al., 2008). Complexity is measured by evaluating the amount of information carried by a biological series, based on entropy-derived non-linear indices: the larger is the information, the greater is the complexity (Porta et al., 2001).

Entropy-derived indices, such as approximate entropy, sample entropy, corrected conditional entropy (CCE) and Shannon entropy (SE) have been proposed (Porta et al., 2001). Although a detailed description of the mathematical basis of these measures is beyond the scope of this review, we will briefly describe the key aspects of SE, Conditional Entropy (CE), and CCE, which have been applied to provide information on cardiac complexity in normal and pathological sleep.

#### Shannon entropy (SE)

SE evaluates the complexity of patterns distribution of length L, RR_L_ = {RR_L_(i) = (RR(i), RR(i − 1), …, RR(i-L + 1)), i = 1, …, N-L + 1)}, by describing the shape of this distribution [SE(L) describes the shape of the distribution of RR_L_]. When SE(L) is calculated with L = 1, it depends on the shape of the distribution of the heart period time series. When SE(L) is large, the pattern distribution is flat, meaning that all the patterns are equally distributed and the amount of information carried by the series is highest. On the opposite, when SE(L) is small, the pattern distribution is not flat but characterized by specific shapes (i.e., Gaussian or skewed distribution), suggesting that some patterns are more present while others are less present or absent (Porta et al., 2001).

#### Conditional entropy (CE) and corrected conditional entropy (CCE)

CE measures the quantity of information carried by the current RR sample when the previous samples are known. In other words, CE corresponds to the difficulty in predicting future values of RR intervals based on past values of the same series. CE is 0 when future values of RR are completely predictable given RR past values and it is equal to SE(1) when the knowledge of past values of RR is not helpful to reduce the uncertainty of future RR values. However, because the estimation of CE is biased (CE became unreliable as a function of L, decreasing very fast toward 0 with L independently of the ability of past values of RR to predict future RR samples), CCE was designed to overcome this mathematical limitation. CCE decreased to 0 when new sample is completely predictable, remained to the maximum value [i.e., SE(1)] when the new sample is fully unpredictable and showed a minimum when the knowledge of past values was helpful to reduce the uncertainty associated to future values (Porta et al., 2007a).

From CCE, it is possible to derive an index of regularity, Ro (obtained by dividing CCE by the Shannon entropy), which is bounded between 1 (maximum regularity, lowest complexity) to 0 (lowest regularity, maximum complexity).

## Heart rate variability in wake/sleep states

The interaction between ANS and sleep is complex, bidirectional and regulated by several different factors. In fact, changes in ANS regulation can profoundly affect sleep onset and sleep homeostasis and, on the opposite, modifications of physiological sleep can impinge upon autonomic cardiovascular regulation. It is well known that sleep is a complex phenomenon in which autonomic cardiac control fluctuates between sympathetic and parasympathetic predominance, mainly according to the transition to different sleep stages (wakefulness, NREM and REM). Somers and colleagues showed that the cardiovascular system is strongly affected by the sleep stage: in fact, from N1 to N3, the stage of highest neural synchronization, a gradual decrease is observed in HR, BP and MSNA, with minimum values reached during N3, also called “quiet sleep” (Somers et al., 1993). REM sleep, however, is characterized by an opposite behavior, with a sort of “activation” of cardiovascular system to levels sometimes higher than wakefulness. Thus, the transition from NREM to REM is accompanied by a significant increase of HR, BP, and MSNA, and, more interestingly, not stable but with continuous fluctuations of the cardiovascular system, suggesting that cardiovascular control is very complex and influenced by several factors during this sleep stage.

Interestingly, animal studies showed that during REM sleep, sympathetic outflows from different sources (i.e., renal and lumbar sympathetic outflows) may change independently from each other, supporting the hypothesis that a differentiate control of sympathetic outflows could be important for arterial pressure regulation (Yoshimoto et al., 2011).

So far, several studies investigating the change of autonomic cardiovascular control by means of HRV during wake and different sleep stages provided fundamental information regarding the relation between autonomic fluctuations and the shift to different sleep stages (Vanoli et al., 1995; Vaughn et al., 1995; Elsenbruch et al., 1999; Crasset et al., 2001; Trinder et al., 2001). According to the changes of HR, BP and MSNA, transition from wake to NREM sleep is associated with a gradual increase in parasympathetic modulation, expressed by an increased HF component and a decreased of LF component of HRV (Cajochen et al., 1994; Busek et al., 2005). Although the meaning of LF oscillation is someway still debated, the decrease of LF rhythm together with the changes in MSNA and BRS seem to suggest a global decrease of sympathetic modulation from wake to NREM sleep.

On the opposite, from NREM to REM sleep a significant reduction in total HRV together with a shift of sympatho-vagal balance toward a vagal withdrawal and a possible sympathetic predominance has been reported (Berlad et al., 1993; Baharav et al., 1995; Versace et al., 2003).

Beyond these general considerations, it is worth noting that several factors can influence autonomic control during sleep stages, such as the stage preceding the change and the sleep cycle time point. In fact, it has been suggested that the degree of increased sympathetic modulation during REM sleep depends on the previous sleep stage, with significantly higher values of LF/HF ratio during N2 preceding REM sleep compared to N2 preceding N3, supporting the hypothesis that autonomic control varies not only according to the sleep stage but also in relation to the preceding and following stages (Busek et al., 2005).

As to the sleep cycle, it has been reported that REM sleep at the end of the night is characterized by an increased sympathetic modulation compared to REM sleep that occurs during the first part of the night. From a clinical point of view, this fact can be a potential link between increased sympathetic drive, REM sleep and the incidence of cardiovascular events in the early morning (Muller et al., 1995; Scholz et al., 1997; Verrier and Josephson, 2009).

The evaluation of spontaneous baroreflex sensitivity (BRS) revealed important information on the cardiac and vascular interaction through baroreflex circuit during sleep. In fact, it has been observed that during REM sleep, BRS is higher compared to nocturnal wakefulness (Monti et al., 2002). During the first sleep cycle, BRS is higher during NREM compared to wakefulness and REM, while, during the last one, BRS is increased during REM compared to NREM, thus suggesting that the efficacy of baroreflex control in blunting REM sympathetic drive is higher during the late sleep cycles (Legramante et al., 2003). Interestingly, this phenomenon is even more evident in hypertensive patients, in which the blunted BRS and the increase of sympathetic modulation are greater at the end of the night, possibly implicating the occurrence of cardiovascular events (Drager et al., 2009).

In addition to classic HRV analysis, the application of non-linear entropy-derived measures revealed important changes of autonomic cardiovascular complexity peculiar for each sleep stage. However, due to the large amount of non-linear methods and experimental protocol differences, conclusive results are still lacking.

**Figure 2:**
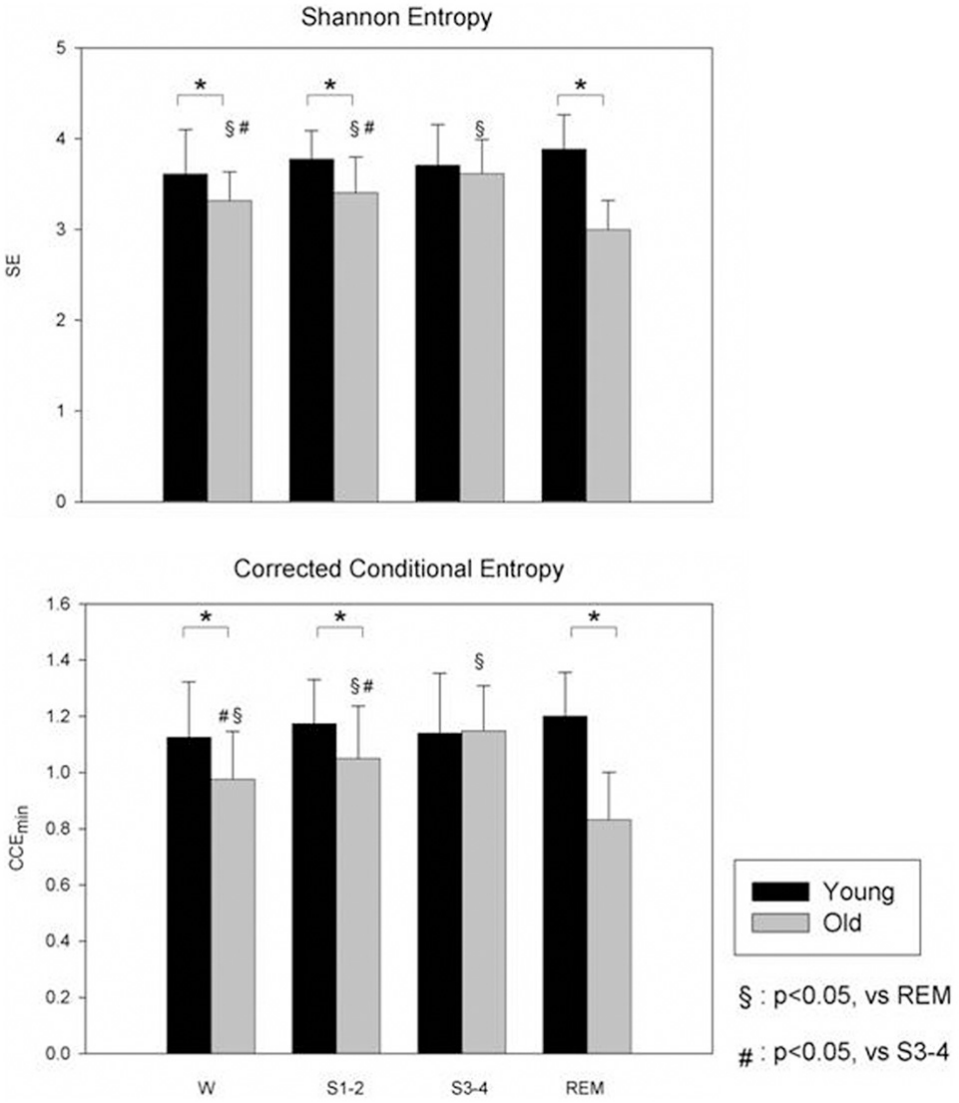
**Non-linear analysis of HRV during different sleep stages in young (black bars) and old (gray bars) subjects during wake (W) and different sleep stages.** Compared to young, the old group have a lower SE. CCE is significantly reduced in old group compared to young and this reduction was more evident during REM sleep (from Viola et al., 2011; with permission). SE, Shannon Entropy; CCE, Corrected Conditional Entropy. ^*^p < 0.05, Young vs. Old.

For instance, previous data showed that NREM sleep is characterized by an increased Sample Entropy while during REM sleep controversial results have been reported, from an increase of Approximate Entropy to a decrease of Sample Entropy (Virtanen et al., 2007; Vigo et al., 2010). A recent study by Viola and colleagues showed that CCE and SE were significantly lower during REM sleep compared to wake and NREM sleep (Viola et al., 2011), and this reduction was more evident in aged people. These data suggested the hypothesis that in aged people, REM sleep more than NREM sleep could be considered a stage of reduced complexity of cardiovascular control, thus, a stage of potential increased cardiovascular risk (see Figure 2).

However, it is worth noting that NREM sleep is not a stable phenomenon; indeed it is punctuated by the occurrence of arousals, which represent transient episodes of cortical and autonomic activation. Moreover, besides the conventional arousals, characterized by fast EEG frequencies (within the alpha, 8–12 Hz, and beta, >16 Hz range), other EEG phasic patterns, such as K-complexes and bursts of slow waves are associated with an activation of vegetative and somatomotor functions (Ferini-Strambi et al., 2000; Ferri et al., 2000; De Carli et al., 2004; Halász et al., 2004). Therefore, both fast and slow EEG arousal components represent a certain degree of cerebral activation reverberating upon the ongoing autonomic activity (Terzano et al., 1985; Parrino et al., 2012). During NREM sleep, arousal fluctuations appear with a pseudo-rhythmic modality, recurring about every 20–40 s. This arousal rhythm, defined under the term of cyclic alternating pattern (CAP) (Terzano et al., 1985, 1988), represents a condition of sustained arousal instability.

CAP alternates with phases of stable sleep (non-CAP, NCAP), characterized by rare and randomly distributed arousal-related phasic events. Different studies have shown that CAP and NCAP are accompanied by significant changes of HRV parameters both in adults and children (Ferini-Strambi and Smirne, 1997; Ferini-Strambi et al., 2000; Ferri et al., 2000). In particular, during a CAP phase an increase in LF and LF/HF ratio has been observed, suggesting that during arousal instability the sympatho-vagal balance is shifted toward a sympathetic predominance (Ferini-Strambi and Smirne, 1997; Ferri et al., 2000). Moreover, the CAP-related increase in sympathetic modulation was higher than the mean value expressed by the corresponding sleep stage as a whole (Ferri et al., 2000). Interestingly, it has been demonstrated that BRS was higher during CAP than NCAP sleep, and it was similar during CAP and REM sleep (Iellamo et al., 2004). The analysis of the relationships between autonomic functions and CAP/NCAP phases could represent a more sensitive tool for the investigation of the effect of sleep disorders on HRV.

## HRV and sleep disorders

In the last decades, a growing interest has been focused on sleep disorders, mainly due to their epidemiological and clinical relevance and to their possible association with cardiovascular diseases. The International Classification of Sleep Disorders classified sleep disorders into several categories, such as (1) insomnias, (2) SDB, (3) hypersomnias, (4) parasomnias, and (5) sleep related movement disorders.

It is worth noting that most sleep disorders are characterized by important modifications of physiological sleep, and often by signs and symptoms of sleep loss, which have been demonstrated to be independent risk factors for cardiovascular morbidity and mortality (Wingard et al., 1982; Gallicchio and Kalesan, 2009; Cappuccio et al., 2010; Redline and Foody, 2011). It has been suggested that autonomic cardiovascular control could be importantly implicated as a potential physiopathological link between sleep disorders and their physiological consequences.

This section will briefly explore HRV changes in three pathological conditions, i.e., SDB, Insomnia, and Sudden Unexpected Death in Epilepsy, which are characterized by noteworthy alterations of cardiovascular autonomic regulation and increased cardiovascular risk.

### HRV in sleep disordered breathing

SDB is a group of diseases characterized by significant alterations of breathing during sleep, which cause fragmentation of physiological sleep, symptoms of sleep deprivation and altered gas exchanges during the night. The most common SDB is Obstructive Sleep Apnea (OSA), which has a prevalence of 2–4% in middle age population.

OSA is associated with repetitive episodes of apneas during sleep, with partial or complete collapse of the upper airway during inspiration; thus, the increased resistance in the upper airways, caused by the collapse of the pharynx and hypopharynx muscles, lead to paradoxical thoracic and abdominal movements in order to overcome the airway obstruction. The frequent episodes of apneas have three main effects: hypoxia and hypercapnia due to gas exchange alterations, sleep fragmentation with repetitive arousal and, finally, irritability, daytime sleepiness and altered cognitive performance.

The clinical relevance of OSA is related to its strong association with obesity, hypertension, and increased cardiovascular risk (Kales et al., 1984; Bliwise et al., 1988; Hung et al., 1990; Somers et al., 1995; Narkiewicz and Somers, 2001). Although the pathophysiological factors linking OSA and cardiovascular risk are not completely understood, several evidences support the hypothesis that sleep fragmentation and intermittent hypoxia cause a chronic hyperactivation of the sympathetic nervous system, a key component of the progression to cardiovascular disease. In addition, endothelial dysfunction and activation of inflammatory cascade have been described in OSA patients, possibly mediated by the activation of the SNS.

OSA must be suspected in patients with snoring, obesity, resistant hypertension and in patients with signs and/or symptoms of sleep loss (fatigue, hypersomnolence, daytime sleepiness, cognitive impairment etc.). The diagnosis of OSA is confirmed in the presence of apneas (defined as a stop in airflow of at least 10 s) and hypopneas (reduction of 50% of the flow, with an oxygen desaturation of >4% and lasting at least 10 s). The number of apneic events is calculated as the apnea/hypopnea index (AHI), i.e., the total amount of apneas and hypopneas per hour of sleep. An AHI lower than five identifies a normal subjects, an AHI between 5 and 15 identifies mild OSA, an AHI between 15 and 30 a moderate OSA and an AHI greater than 30 a severe OSA (Somers et al., 1995).

The repetitive episodes of apneas have important hemodynamic and cardiovascular consequences, which result evident both during nighttime and daytime. Subjects with OSA usually have higher resting HR and BP (Vanninen et al., 1996). The autonomic consequences of OSA mainly involve chemoreflex and baroreflex regulation, with a global shift of the sympatho-vagal balance toward a sympathetic predominance and a blunted parasympathetic control (Narkiewicz et al., 1998), evident either during wake, and during night. Namely, during apneic events, the physiological inhibition of sympathetic activity by lung inflation is lacking, causing a significant sympathetic activation which is responsible for increased in BP and changes in HR. Sympathetic activity is highest at the end of an apnea, when hypoxia and hypercapnia reach their maximum levels; after the upper airways re-opening, it is possible to observe a large raise in blood pressure, which activates baroreflex control and induces a temporary withdrawal of sympathetic overactivity (Narkiewicz and Somers, 2001). The analysis of HRV revealed that during daytime, moderate to severe OSA patients were characterized by increased cardiac sympathetic modulation compared to mild-OSA and controls (Narkiewicz et al., 1998). Compared to controls, OSA subjects are characterized by a lower total variability and a possible shift of the sympatho-vagal balance toward a sympathetic predominance and a vagal withdrawal, as shown by the increase of LF component and LF/HF and the decrease of HF component (Narkiewicz and Somers, 2003; Smietanowski et al., 2006; Kesek et al., 2009) either during wakefulness and during nighttime (Shiomi et al., 1996; Vanninen et al., 1996).

It is important to underline a limitation of HRV application to sleep studies in OSA at this point. Indeed, the analysis of HRV during sleep is limited just by the presence of repetitive apneas, leg movements, or arousals, which artificially modify HRV analysis, introducing a “rhythmic” biological noise able to alter autonomic cardiovascular oscillations. This problem was highlighted by few studies that found that a higher AHI was associated with a higher vagal modulation during NREM sleep (da Silva et al., 2009) and, in contrast to the general expectations, in severe OSA patients, a decreased sympathetic regulation during REM sleep was observed (Gula et al., 2003). In fact, severe OSA induced an important modification in breathing pattern, which could have *per se* been a relevant confounding factor able to impinge upon HRV rhythmical oscillations. For this reason, cardiovascular autonomic assessment in severe OSA patients can importantly be affected by non-neural oscillations related to the continuous episodes of apnea which could thereby alter HRV analysis (Wang et al., 2008); this factor must be taken into account when analyzing PSG data and interpreting the results. To this regard, in a recent paper assessing HRV during sleep in patients with Brugada syndrome diagnosed with or without SDB, ECG recordings derived from polysomnographic studies were analyzed, carefully avoiding periods with apneas/hypopneas and considering only ECG segments associated with stable and regular breathing (Tobaldini et al., 2013). This approach allowed the observation that Brugada syndrome, a rare but life- threatening disease characterized by ventricular arrhythmias and sudden cardiac death, more frequent during nighttime, was associated with an impaired autonomic cardiovascular control in the presence of comorbid SDB (Tobaldini et al., 2013).

The gold standard therapy for OSA is the application of continuous positive airway pressure (CPAP), a device able to maintain open the upper airways during sleep by inflating a positive pressure airflow, preventing the repetitive airway collapses as previously mentioned (Patel et al., 2003). CPAP therapy is able to improve cardiovascular outcome (Marin et al., 2005), with a significant reduction of arterial pressure (Faccenda et al., 2001), inflammatory markers, insulin resistance (Brooks et al., 1994), and coagulation factors (Phillips et al., 2012). As to autonomic effects of CPAP, a pioneer study by Somers and colleagues showed that CPAP is able to acutely affect ANS, with a significant decrease of MSNA during wake and sleep (Narkiewicz et al., 1999). Interestingly, even one night of CPAP treatment was able to affect HRV with a reduction of sympathetic modulation and an improvement of baroreflex control (Bonsignore et al., 2006; Kufoy et al., 2012). Longer CPAP treatments revealed positive effects on hemodynamic and metabolic variables, such as an improvement of arterial stiffness, a reduction of inflammatory response (Arias et al., 2008; Dorkova et al., 2008) and a decrease of platelet aggregation (Shimizu et al., 2002). However, the effects of longer CPAP treatments showed contrasting results and conclusive results on its consequences on HRV are still lacking. For instance, a reduction of the LF component and the LF/HF ratio, have been described, likely to be related to an improvement of chemoreflex and baroreflex responses (Roche et al., 1999; Khoo et al., 2001). Very recently, it has been described that long term CPAP (2 years treatment) is able to improve the coupling between parasympathetic modulation and delta wave sleep (Jurysta et al., 2013), suggesting a positive effect of this therapy on central and peripheral oscillations.

### HRV in insomnia

Insomnia is a sleep disorder characterized by an important inability to fall asleep, to stay asleep or to wake up too early, causing daytime sleepiness, fatigue, mood alterations, and memory impairment. Insomnia is one of the most common sleep disorders and it can be classified into (1) comorbid insomnia, i.e., associated with other diseases, either physical and mental, (2) primary insomnia (PI), i.e., unrelated to any other disease, and (3) chronic (or long-standing) insomnia.

Apart from the relevant effects on day-life activity, the importance of insomnia as a potential modifiable risk factor for the development of cardiovascular diseases has been recently underlined (Spiegelhalder et al., 2010; Redline and Foody, 2011).

The assessment of ANS control in insomniac patients revealed interesting results. A pioneer study by Bonnet and colleagues showed a significant increase of the LF component and a decrease of the HF component of HRV in insomniacs compared to healthy subjects during sleep; these data suggested for the first time that insomnia is characterized by a predominant sympathetic modulation not only during wake but also across sleep stages (Bonnet and Arand, 1998).

These results have been confirmed by successive studies, which showed that PI patients exhibit a constant sympathetic overactivity during night (de Zambotti et al., 2013) and a marked reduction of vagal modulation, indicated by the decrease of the HF component of HRV during the night (Spiegelhalder et al., 2011; Yang et al., 2011).

These data support the hypothesis that in PI patients, sympatho-vagal balance is shifted toward a sympathetic predominance. Furthermore, the analysis of complexity indexes using entropy derived measures showed a considerable decrease in complexity during nighttime compared to healthy subjects, thus suggesting a possible link with cardiovascular diseases (Yang et al., 2011).

It is worth noting that PI patients do not only have an impaired autonomic cardiac modulation, but also an altered coupling between HRV and delta sleep. In fact, analyzing the relationship between the HF component and EEG delta power, PI patients showed a decreased HF-delta EEG coherence with respect to controls, thus suggesting a significant change in the interaction between central and peripheral drives (Jurysta et al., 2009).

In summary, insomnia is a very common sleep disorder, relevant for its prevalence over general population and for its clinical consequences. An altered autonomic cardiovascular control has been described in insomniac patients, who show a shift of the sympatho-vagal balance toward a predominance of sympathetic modulation both during wake and night; this alteration could be responsible for increased risk of cardiovascular diseases.

### HRV, sleep, and sudden unexpected death in epilepsy (SUDEP)

Epilepsy is a brain disorder characterized by an enduring predisposition to generate seizures. Seizures are paroxysmal transient disturbances of brain functions that may be manifested as episodic impairment or loss of consciousness, abnormal motor phenomena and psychic or sensory disturbances. Moreover, seizures can induce a perturbation of the ANS; indeed, neurovegetative symptoms such as cardiovascular and respiratory changes, gastro-intestinal, cutaneous, and genito-urinary manifestations, frequently occur during epileptic seizures (Baumgartner et al., 2001; Leutmezer et al., 2003). Finally, it has been shown that also sub-clinical epileptic discharges may be associated with autonomic instability (Brotherstone and McLellan, 2012).

In epileptic patients, especially in those with pharmacoresistant epilepsy, the risk of sudden unexpected death (SUDEP) is 24–40 times higher with respect to the general population (Ficker et al., 1998; Mohanraj et al., 2006). SUDEP is considered to be the result of a peri-ictal concurrence of a number of predisposing and precipitating factors (Nobili et al., 2011); nevertheless SUDEP is primarily a sleep related phenomenon and sleep related seizures seem to be an independent risk factor for SUDEP (Lamberts et al., 2012). Previous studies have shown decreased HRV in chronic epileptic patients suggesting that this might play a role in the pathophysiology of SUDEP (Tomson et al., 1998; Ansakorpi et al., 2000; Ronkainen et al., 2005). There are many reports suggesting that the autonomic changes observed in epileptic patients are mainly evident during nocturnal sleep. Indeed, patients (both adult and children) with focal epilepsy exhibit a higher reduction of HRV during night-time with respect to control subjects, indicating that the sleep period in epileptic patients might be more at risk of developing alterations of autonomic heart control (Ferri et al., 2002; Ronkainen et al., 2005; Persson et al., 2007). In a recent study, conducted in a population of drug resistant epileptic children with different clinical syndromes, authors found a striking reduction in vagal modulation during slow-wave sleep and a small autonomic modulation capacity (Jansen et al., 2011).

There is no definitive interpretation whether the observed autonomic changes in epileptic patients are due to the recurrence of seizures, the interictal epileptic discharges, and/or to the drug treatment. Seizures and periodic epileptic discharges, increasing arousal fluctuations during NREM sleep, might lead to a chronic stimulation of the ANS which are reflected by changes in HRV parameters. The relevant role of seizures and epileptic discharges on HRV change seems to be confirmed by the observation that, in drug resistant patients, HRV improves after epilepsy surgery especially in those with a positive outcome (Hilz et al., 2002; Dütsch et al., 2004; Persson et al., 2005). Also antiepileptic drugs could influence the autonomic state; in particular carbamazepine and polytherapy seem to reduce HRV (Persson et al., 2003; Yildiz et al., 2011). A recent systematic review of case-control studies, enrolling patients with different epilepsy syndromes and from infancy to adulthood, confirmed that epileptic patients present lower HF values, indicating impaired vagal control associated with increased cardiovascular risk and arrhythmias (Lotufo et al., 2012). Moreover a trend for lower LF ratios was identified in epileptic patients using pharmacotherapy.

In conclusion, the reduction of HRV that has been found in patients with epilepsy seems to be more pronounced during the night, thus affecting the circadian HRV. Further studies are needed to assess the possible association between altered HRV and risk of sudden death during sleep in epileptic patients.

## Conclusions

In summary, sleep is a complex biological phenomenon regulated by different biological pathways. Cardiovascular autonomic control plays a key role, varying among the transition to different sleep stages. In addition, the sleep-autonomic link has to be considered bidirectional: in fact, autonomic changes can importantly alter sleep regulation and, on the other side, sleep disturbances can profoundly alter the physiological cardiac autonomic modulation. Nowadays, an increasing prevalence of sleep disorders such as SDB and neurological sleep related disturbances have been described. The assessment of autonomic cardiovascular control using classical linear and more recent non-linear analysis of HRV have been widely used as non-invasive tools to provide important information on autonomic changes in physiological and pathological sleep.

### Conflict of interest statement

The authors declare that the research was conducted in the absence of any commercial or financial relationships that could be construed as a potential conflict of interest.

## Abbreviations

AHI: apnea/hypopnea index
ANS: autonomic nervous system
BP: blood pressure
BRS: baroreflex sensitivity
CAP: cyclic alternating pattern
CCE: corrected conditional entropy
CE: conditional entropy
CPAP: continuous positive airway pressure
EEG: electroencephalogram
HF: high frequency
HR: heart rate
HRV: heart rate variability
LF: low frequency
MSNA: muscle sympathetic nerve activity
NCAP: non-CAP
NREM: non-REM
NU: normalized units
OSA: obstructive sleep apnea
PI: primary insomnia
REM: rapid eye movement
Ro: regularity index
SDB: sleep disordered-breathing
SE: Shannon entropy
SUDEP: sudden unexpected death in epilepsy
SWS: slow wave sleep
VLF: very low frequency
VLPO: ventrolateral preoptic nucleus.
